# Unraveling human crowd dynamics through the foot tracking of pedestrians

**DOI:** 10.1126/sciadv.adw2688

**Published:** 2025-06-25

**Authors:** Yi Ma, Zhipan Niu, Meng Shi, Wei Xie, Zuoan Hu, Yidong Wei, Tian Zeng, Eric Wai Ming Lee

**Affiliations:** ^1^ Institute for Disaster Management and Reconstruction, Sichuan University, Chengdu 610065, China.; ^2^ School of Computer Science, South-central Minzu University, Wuhan 430074, China.; ^3^ School of Transportation and Logistics, Southwest Jiaotong University, Chengdu 610065, China.; ^4^ Department of Civil and Environmental Engineering, The Hong Kong Polytechnic University, Hong Kong, China.; ^5^ Department of Architecture and Civil Engineering, City University of Hong Kong, Hong Kong, China.

## Abstract

Movement of pedestrian crowds is ubiquitous in human society. However, it is unclear what dynamical regimes pedestrian crowds can exhibit at different crowd densities, how pedestrians move in these different dynamical regimes, and in which dynamical regime the movement synchronization of pedestrians is most likely to occur. Here, we conducted a unidirectional crowd movement experiment, in which we tracked the movement of pedestrian crowds through foot tracking. We find experimentally that pedestrian crowds can exhibit three distinct dynamical regimes (free regime, slow-moving regime, and jammed regime) depending on the crowd density. In the free regime, pedestrians’ movement is not constrained; in the slow-moving regime, pedestrians’ speed is constrained, but pedestrians’ movement direction in each step is not influenced; and in the jammed regime, both pedestrians’ speed and movement direction in each step are constrained. We also demonstrate that pedestrians are most likely to synchronize their movements spontaneously at the onset of jamming. Our findings provide important insights into crowd dynamics.

## Teaser

A crowd dynamics experiment reveals how pedestrian crowds move in different dynamical regimes.

## INTRODUCTION

Crowd dynamics (i.e., the issue of how pedestrian crowds move) is a captivating research topic. Over the past decade, crowd dynamics has received much attention in the fields of statistical physics, human behavior, and transportation engineering (*1*–*24*), because its understanding is crucial for the prediction of crowd movements (*25*), the prevention of crowd disasters (*26*–*29*), the design and planning of urban infrastructures (*30*), and the realization of future social robots (*31*).

Despite this importance, our understanding of crowd dynamics remains unexpectedly incomplete. The movement of pedestrians is essentially completed by the pedestrians’ foot. However, in existing empirical studies of crowd dynamics, the tracking and analysis of pedestrian crowd movement are usually based on pedestrian head tracking (*2*–*9*) rather than pedestrian foot tracking. Although pedestrian head tracking is sufficient for measuring some fundamental physical quantities (e.g., density, velocity, and flow rate) and elucidating some movement characteristics (e.g., density-velocity and density-flow rate relations) (*1*, *4*–*8*) and self-organized phenomena (e.g., lane formation) (*9*, *10*) of pedestrian crowds, pedestrians’ motion behavior cannot be completely captured through head tracking. For example, the accurate movement direction, speed, stop time, step length, and step frequency of pedestrians in each natural step are difficult to precisely measure through head tracking. As a result, the issue of crowd dynamics is difficult to address fully. The main barrier to exploring crowd dynamics from a foot perspective is how to precisely and synchronously track multipersonal foot trajectories in dense crowds. It is not difficult to track the foot motion trajectory of a single isolated person or persons in ordered arrangements (*32*–*34*). However, in dense two-dimensional crowds, it is considerably challenging to precisely and synchronously track the foot motion trajectories of intracrowd pedestrians, because pedestrians’ feet are easily occluded by neighbors’ bodies. This is why, until now, few studies have explored crowd dynamics through pedestrian foot tracking.

Although pedestrian foot tracking in crowds is challenging, it could enable a more complete understanding of crowd dynamics. In particular, pedestrian foot tracking enables the precise capture of the dynamics of pedestrians in each natural step. This allows us to deeply explore many essential problems regarding pedestrian crowd dynamics, such as how pedestrians move in each natural step and how self-organized synchronization emerges. Understanding these problems is crucial for managing crowd flow and preventing crowd disasters. For example, a sufficient understanding of the emergence of self-organized synchronization of pedestrians can provide important guidance for the prevention of pedestrian synchronization–induced wobbling disaster in large-spanned pedestrian facilities (e.g., footbridges) (*35*, *36*). In addition, understanding these problems is crucial for the development of future human-shaped bipedal walking robots (*37*), because knowledge of human footsteps is indispensable for gait design. Given this importance, it is necessary to explore crowd dynamics through pedestrian foot tracking.

Despite the importance of pedestrian foot tracking, few studies have attempted to explore crowd dynamics through pedestrian foot tracking (*34*, *38*–*42*). We noted that in these few studies exploring crowd dynamics through pedestrian foot tracking, foot tracking was usually restricted to pedestrians walking in a line (*34*, *38*–*41*). In this setup, the movement of each pedestrian was one-dimensional and influenced only by the preceding and following pedestrians. However, in the real world, most pedestrian movements are two-dimensional. The dynamics of pedestrian crowds in the two-dimensional space are more complex than those in the one-dimensional space, because pedestrian movements in the two-dimensional space have a higher degree of freedom and are influenced not only by the preceding and following pedestrians but also by those to the left and right. Foot tracking on two-dimensional pedestrian crowds is rare. Recently, Tomaru *et al.* (*42*) made an important attempt to track pedestrian footsteps in bidirectional flows using inertial measurement units placed on pedestrians’ feet. This method greatly inspires the empirical study of crowd dynamics from a foot perspective. The inertial measurement units could record the stop and move states of pedestrians’ feet; however, it would be difficult to directly record the full motion trajectories of pedestrians’ feet. Overall, up to now, there has been no attempt to deeply explore the dynamics of two-dimensional pedestrian crowds through the synchronized tracking of pedestrian foot motion trajectories.

In dense two-dimensional crowds, synchronously tracking the foot motion trajectories of intracrowd pedestrians is considerably challenging because pedestrians’ feet are easily occluded by neighbors’ bodies. In previous empirical studies of crowd dynamics, tracking via camcorders set over the heads of pedestrians was the most widely used tracking method. However, this method is not applicable for tracking the foot motion trajectories of two-dimensionally distributed pedestrians because of the body occlusions between pedestrians, especially at high densities. Similarly, three-dimensional optical motion capture techniques have difficulty overcoming this occlusion issue. Although wearing mini wireless positioning devices on pedestrians’ feet provides the possibility for tracking the foot motion trajectories of two-dimensionally distributed pedestrians, the stability of the wireless positioning signal and the accuracy of the corresponding positioning are still influenced by the occlusion of the bodies of pedestrians. Paving sensors on the floor to track the foot motion trajectories of pedestrians is also less feasible because the position signal of the foot is lost when pedestrian’ foot leaves the paved pressure sensors on the floor to take a step.

**Fig. 1.**
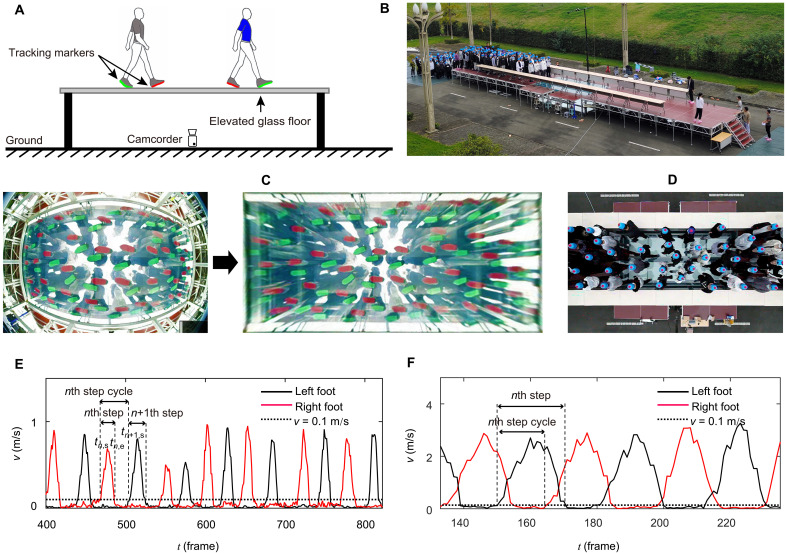
Experimental design. (**A**) Idea illustration of pedestrian foot tracking. (**B**) Photo of the constructed elevated experimental platform. (**C**) Schematic illustration of the synchronous tracking of pedestrian foot motion from the ground with a bottom-up viewing angle when pedestrians walk through the transparent observation area in the middle of the experimental platform. The left image shows the raw image recorded by the fish-eye camera under the glass floor, and the right image is the result after distortion correction. (**D**) Schematic illustration of the tracking of pedestrian head motion with an overhead viewing angle. (**E**) Monitored dynamic variation in the speed of a pedestrian’s feet with time in the jammed regime. (**F**) Monitored dynamic variation in the speed of a pedestrian’s feet with time in the free regime.

In this study, we conducted a large-scale unidirectional crowd movement experiment to study crowd dynamics. Unlike many previous large-scale crowd movement experiments, in which the motion tracking of pedestrian crowds was based on pedestrian head tracking (*2*–*9*), in our experiment, we tracked the movement of pedestrian crowds through pedestrian foot trajectory tracking. As shown in Fig. 1, pedestrian foot trajectory tracking was realized through an elevated pedestrian corridor built with a glass floor. When pedestrians walked through the elevated glass floor, pedestrian foot movement could be clearly observed through the glass floor from the ground with a bottom-up viewing angle (see Materials and Methods for complete details about the experiment). The tracking of pedestrian foot trajectory enabled us to accurately measure the movement direction, speed, stop time, step length, and step frequency of pedestrians in each natural step and to precisely capture the occurrence of movement synchronization of pedestrians. Through the experiment, we address the following questions: (i) What dynamical regimes can pedestrian crowds exhibit at different crowd densities? (ii) How do pedestrians move in these different dynamical regimes? (iii) In which dynamical regime is the movement synchronization of pedestrians most likely to occur and why?

## RESULTS

### Evidence of the existence of three dynamical regimes of pedestrian crowds

We conducted a unidirectional crowd movement experiment, in which we tracked the movement of pedestrian crowds through pedestrian foot tracking. As shown in Fig. 1, pedestrian foot tracking was realized through an elevated pedestrian corridor. The floor of the middle segment of the corridor was a whole piece of specially designed toughened laminated glass plate. That is, the floor of the middle segment of the corridor was fully transparent. This key design allowed us to monitor the pedestrians’ foot motion from the ground with a bottom-up viewing angle when pedestrians walked through this area, as shown in Fig. 1C. We conducted crowd movement experiments in the corridor using pedestrian crowds of different densities. Besides, to regulate the crowd density in the corridor, we set a bottleneck at 3.6 m from the transparent observation area on one side of the transparent observation area. Then, we performed 40 runs of experiments using different crowd sizes and bottleneck widths, including using 20, 30, 40, 50, 60, 70, 80, 90, 100, 110, and 120 participants in the case of a bottleneck width of 2.4 m, as well as using bottleneck widths of 2.2, 2.0, 1.8, 1.6, 1.4, 1.2, 1.0, 0.8, 0.7, 0.6, 0.5, and 0.4 m in the case of 120 participants. We recorded the pedestrians’ foot motion with a bottom-up viewing angle through a high-resolution camcorder under the transparent glass floor in the experiments (see Materials and Methods for details about the experiment). Then, we extracted the pedestrians’ foot motion trajectories from the recorded video clips and measured the pedestrian motion parameters, including the moving speed of each foot of each pedestrian at each moment, the crowd density and interpersonal distance at the beginning of each natural step of each pedestrian, and the flow speed, flow rate, step length, step frequency, stop duration, and directional angle of each pedestrian in each natural step (see Eqs. 1 to 9 in Materials and Methods for the detailed measurement methods of these parameters).

**Fig. 2.**
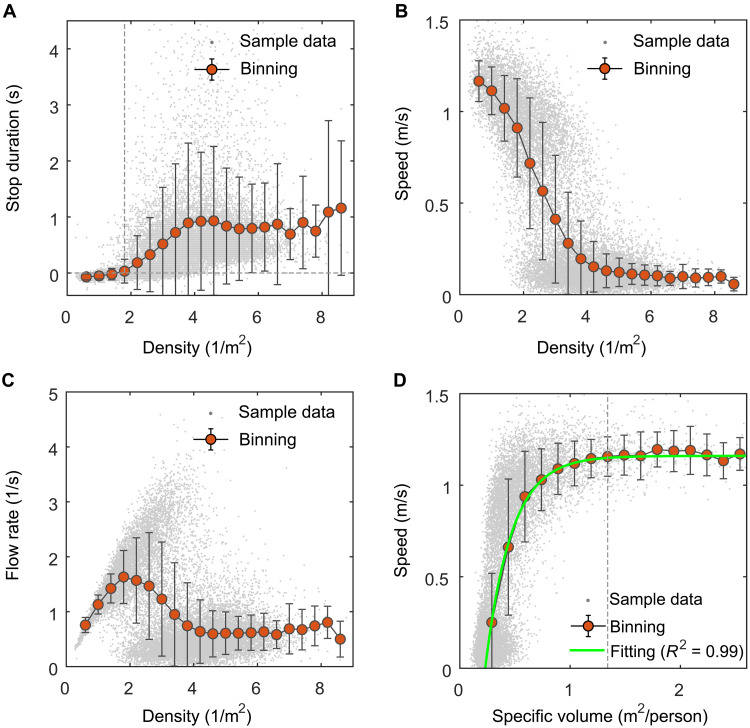
Relationships between pedestrian movement parameters. (**A**) Relationship between crowd density and pedestrian stop duration. The vertical dot line denotes that the crowd density is 1.80 persons/m^2^, and the horizontal dot line denotes that the speed is 0 m/s. Note that the stop duration at low densities was calculated to be a small negative value. This was resulted from the overlap phenomenon of steps on the left foot and right foot. During the walking at low densities, when a foot of a person has not fully touched the ground (i.e., only a part of sole touches the ground) to complete the whole step, the heel of another foot is usually already lifted from the ground to take the next step, resulting in a slight overlap of footsteps on the left foot and right foot and a negative stop duration, as illustrated in Fig. 1F. (**B**) Relationship between crowd density and pedestrian speed. Note that the data points at low and high densities are relatively dense and approximately present the trend of two clusters. They (two clusters) are merged to completely reflect and describe pedestrian dynamics under diverse density conditions. (**C**) Relationship between crowd density and pedestrian flow rate. (**D**) Relationship between specific volume and pedestrian speed. The vertical dotted line denotes that the specific volume is 1.34 m^2^ per person, and the green line is the fitting result. In (A) to (D), a gray point represents raw sample data. The red points represent the means after binning processing. The error bars represent the standard deviation.

We first investigated what dynamical regimes pedestrian crowds can exhibit at different crowd densities based on the experiment. Previously, the dynamical regimes of pedestrian crowds were often roughly divided into the free-flow regime and jammed regime (JR) (*11*). In the jammed regime, pedestrians’ movement is strongly constrained. Therefore, we explored what crowd densities result in the emergence of the jammed regime. To address this question, we linked the stop duration (note that the stop duration refers to the time from the ending moment of a footstep to the starting moment of the next footstep, i.e., *t*_*n*,e_ to *t*_*n*+1,s_ in Fig. 1E) of each pedestrian in each natural step in the experiment with the crowd density at the starting moment of the step (see Eqs. 2 and 8 for the calculation methods of the stop duration and crowd density). The resulting relation diagram is shown in Fig. 2A. The results show that when the crowd density is smaller than ~1.80 persons/m^2^, people’s desired stop duration (i.e., mean stop duration) in each natural step is lower than zero, whereas when the crowd density is greater than 1.80 persons/m^2^, the desired stop duration is greater than zero. This directly indicates that crowd densities greater than 1.80 persons/m^2^ can lead to people being unable to move; that is, the jammed regime emerges. Through the density-speed and density-flow rate fundamental diagrams obtained from our experiment (see Figs. 2, B and C), we find that the identified jamming density (1.80 persons/m^2^) is the same as the peak density of the flow rate, verifying that the peak density of the flow rate is the jamming density of pedestrian crowds, as reported in previous study (*11*).

We further explored the following question: At what crowd densities can pedestrians maintain a free regime (FR)? Note that owing to the high sensitivity of the speed to the density at low densities in the density-speed fundamental diagram (see Fig. 2B), the transition point of the speed from its unconstrained state to constrained state (i.e., the critical point of the free regime) cannot be clearly identified. Therefore, we converted the independent variable from density to specific volume (i.e., the reciprocal of density) and linked the specific volume with the speed. The resulting relation diagram is shown in Fig. 2D. The result clearly shows that when the specific volume is greater than 1.34 m^2^ per person, the speed maintains a relatively steady state of ~1.16 m/s; when the specific volume is smaller than ~1.34 m^2^ per person, the speed is no longer steady and instead starts to decrease continuously with decreasing specific volume. The dynamical regime in which pedestrians can maintain a steady moving state without constraints from neighbors is the free regime, as called in previous studies (*5*, *43*). Note that the specific volume is the reciprocal of density. Therefore, this also means that when the crowd density is smaller than 0.75 persons/m^2^, pedestrians can maintain a free regime.

Notably, the critical point of the free regime is 0.75 persons/m^2^, and the critical point for the jammed regime is 1.80 persons/m^2^. This suggests that there exists an in-between regime between the free regime and the jammed regime (i.e., between 0.75 and 1.80 persons/m^2^). From the density-stop duration and density-speed diagrams shown in Fig. 2 (A and B), we can see that in this in-between regime, pedestrians’ speed decreases, but pedestrians’ desired stop duration in each natural step is lower than zero (i.e., no stop), meaning that pedestrians are not substantively jammed. We refer to this in-between regime as the slow-moving regime (SMR). Therefore, to be precise, pedestrian crowds can exhibit three distinct dynamical regimes (free regime, slow-moving regime, and jammed regime) depending on crowd density. This result is consistent with the result observed in one-dimensional single-file crowd environments (*5*, *34*).

**Fig. 3.**
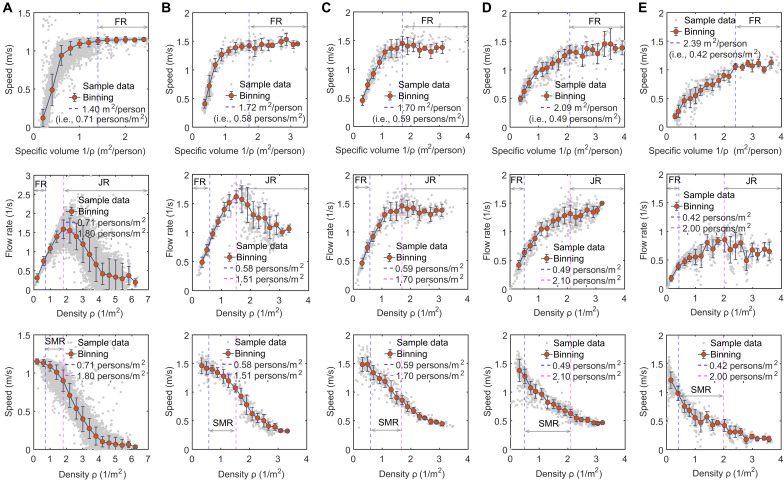
Observation of the free regime (FR), slow-moving regime (SMR), and jammed regime (JR) in the simulation of crowd movement and previous crowd movement experiments. (**A** to **E**) Corresponding observation result in the simulation (note that the simulation model was an improved social force model; see Materials and Methods for the details). In the simulation, the velocity vector of pedestrians was calculated by Eqs. 10 to 12, while the position update of pedestrians was based on Eqs. 13 to 15 (unidirectional crowd flow, bidirectional countercrowd flow, bidirectional cross-crowd flow, and four-directional cross crowd flow). In (A) to (E), a gray point represents raw sample data. The red points represent the means after binning processing. The error bars represent the standard deviation. The blue dotted line represents the observed critical point of the free regime, whereas the red dotted line represents the observed critical point of the jammed regime.

We additionally explored whether the three dynamical regimes can be reflected in the numerical simulations of crowd movement and previous experiments on crowd movement. Accordingly, we performed a simulation of crowd movement based on an improved social force model (see Materials and Methods for details about the adopted model) and an additional empirical analysis based on the public datasets of crowd flow, including datasets of a unidirectional crowd flow, a bidirectional countercrowd flow, a bidirectional cross-crowd flow, and a four-directional cross-crowd flow (see Materials and Methods for details about the source of the datasets). Figure 3 shows the specific volume-speed, density-flow rate, and density-speed relationship diagrams obtained from the simulation and from four public datasets of crowd flows. Each specific volume-speed relation diagram shows that when the specific volume exceeds a certain value (marked by the blue dotted line in each diagram), the speed will maintain a relatively steady state. This dynamical regime is clearly the free regime. From each density-flow rate relationship diagram, we can observe that when the density exceeds a certain value (marked by the red dotted line in each diagram), the flow rate decreases with the density, indicating the emergence of a jammed regime. We marked the critical density of the free regime identified from the specific volume-speed diagram with a blue dotted line in the density-flow rate diagram. We can see that there exists an in-between regime between the free regime and the jammed regime (i.e., between the blue dotted line and the red dotted line in each diagram). From corresponding density-speed diagrams, we can see that in this in-between regime, the speed obviously decreases. This in-between regime is the slow-moving regime. Therefore, the three dynamical regimes can actually be reflected in the numerical simulations of crowd movement and previous experiments on crowd movement.

### Movement characteristics of pedestrians in the three dynamical regimes

**Fig. 4.**
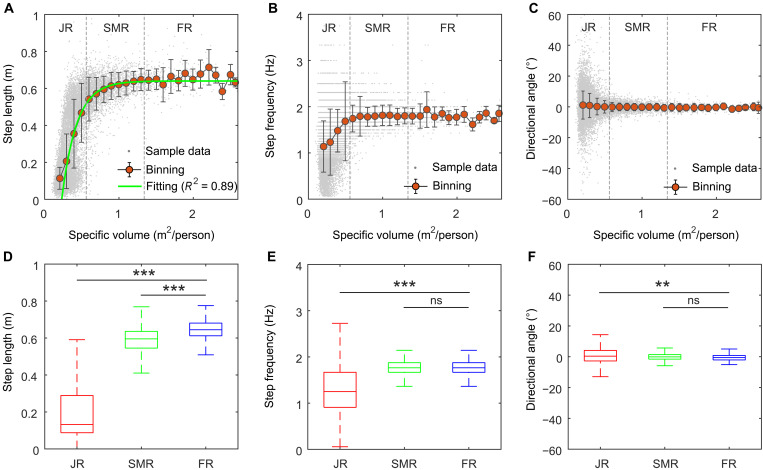
Characteristics of pedestrian step length, step frequency, and directional angle in the free regime, slow-moving regime, and jammed regime. (**A**) Step length versus specific volume. (**B**) Step frequency versus specific volume. (**C**) Directional angle versus specific volume. (**D**) Comparison and statistical analysis of step lengths in the three regimes. (**E**) Comparison and statistical analysis of step frequencies in the three regimes. (**F**) Comparison and statistical analysis of directional angles in the three regimes. In (A) to (C), a gray point represents raw sample data. The red points represent the means after binning processing. The error bars represent the standard deviation. The two vertical dot lines respectively denote that specific volumes are 0.56 and 1.34 m^2^ per person, i.e., the critical points between the free regime, slow-moving regime, and jammed regime. Besides, in (A), the blue line is the fitting result. In (D) to (F), five horizontal lines from lower to upper parts in each box-and-whisker unit represent the minimum, first quartile, median, third quartile, and maximum of the data. Asterisks indicate the statistical significance [***P < 0.001; **P < 0.01; not significant (ns), *P* > 0.05].

In this section, we explore how pedestrians move in these three different dynamical regimes. We first investigated the movement characteristics of pedestrian crowds in the jammed regime. Figure 2B shows that in the jammed regime, pedestrians’ speed sharply decreases. To better understand the movement characteristics of pedestrians in the jammed regime, we linked the step length, step frequency, and directional angle (note that the directional angle refers to the angle between the actual moving direction and the desired moving direction; the desired moving direction refers to the direction parallel to the corridor) of pedestrians in each natural step with the specific volume at the starting moment of the step (see Eqs. 6, 7, and 9 for the measurement methods of the step length, step frequency, and directional angle). The resulting relation diagrams are shown in Fig. 4. Figure 4 shows that in the free regime, the step length, step frequency, and the desired value and standard difference of the directional angle of pedestrians maintain relatively stable states of ~0.64 m, 1.80 Hz, 0°, and 3°, respectively. In the jammed regime, the step length and step frequency are significantly different from the step length [*t* test: *F* = 300.873; *t* = −46.396; df = 15727; *P* < 0.001; 95% confidence interval (CI) = −0.45752 to −0.42043; Cohen’s *d* = 3.34] and step frequency (*t* test: *F* = 89.051; *t* = −14.998; df = 15728; *P* < 0.001; 95% CI = −0.60701 to −0.46669; Cohen’s *d* = 1.10) in the free regime, and both of them show a sharp decreasing trend. Because speed is the product of step length and step frequency, pedestrians’ speed naturally sharply decreases. In addition, we also find that in the jammed regime, the directional angle of pedestrians in each natural step is significantly different from that in the free regime (*t* test: *F* = 103.834; *t* = 2.873; df = 15728; *P* = 0.004; 95% CI = 0.39828 to 2.10939; Cohen’s *d* = 0.21). This finding indicates that in the jammed regime, not only is the pedestrians’ movement speed constrained, but also their movement direction is influenced.

We next explored the movement characteristics of pedestrian crowds in the slow-moving regime. Figures 2D and 4F show that in the slow-moving regime, although the speed of pedestrians is different from that in the free regime, the directional angle of pedestrians is not significantly different from that in the free regime (*t* test: *F* = 0.494; *t* = 1.880; df = 3139; *P* = 0. 060; 95% CI = −0.01178 to 0.56280; Cohen’s *d* = 0.11), indicating that unlike in the jammed regime, both pedestrians’ speed and movement direction are constrained, and in the slow-moving regime, only pedestrians’ movement speed is constrained, whereas pedestrians’ movement direction is not influenced. We also find from Figs. 4 (D and E) that in the slow-moving regime, pedestrians’ step length is significantly different from that in the free regime (*t* test: *F* = 16.807; *t* = −12.143; df = 3139; *P* < 0. 001; 95% CI = −0.07478 to −0.05399; Cohen’s *d* = 0.78), but their step frequency is not significantly different from that in the free regime (*t* test: *F* = 1.757; *t* = −1.465; df = 3139; *P* = 0.143; 95% CI = −0.05655 to 0.00818; Cohen’s *d* = 0.09), suggesting that unlike in the jammed regime, both pedestrians’ step length and step frequency are constrained, and in the slow-moving regime, only pedestrians’ step length is constrained, whereas pedestrians’ step frequency is not influenced. This result also means that in the slow-moving regime, the decrease in pedestrian movement speed essentially results from the decrease in pedestrian step length and is independent of pedestrian step frequency.

Notably, in the slow-moving regime and jammed regime, the variation in pedestrian speed (*v*) with the specific volume (η) overall exhibits exponential behavior (i.e., the speed decreases exponentially with decreasing specific volume) and correspondingly can be approximately described by an exponential equation: $v=1.16\left[1-e^{-4.45\left(\eta -0.23\right)}\right]$ (*R*^2^ = 0.99; sample size: 16), as shown by the green curve in Fig. 2D. Because the specific volume is the reciprocal of density, we can reversely deduce that the relationship between pedestrian speed (*v*) and crowd density (ρ) follows $v=1.16\left[1-e^{-4.45\left(1/\rho -0.23\right)}\right]$ . This equation is exactly the Kladek equation (*44*). In addition, we find that the variation in pedestrians’ step length (*L*) with the specific volume (η) also exhibits exponential behavior and can be approximately described by an exponential equation: $L=0.64\left[1-e^{-4.67\left(\eta -0.23\right)}\right]$ (*R*^2^ = 0.89; sample size: 25), as shown by the green curve in Fig. 4A. In view of this similarity, we tested the correlations between the speed and the step length and between the speed and the step frequency (see fig. S1). The results show that pedestrian speed is strongly linearly correlated with pedestrian step length (*r* = 0.971; *P* < 0.001; sample size: 18,538), whereas it is less strongly correlated with pedestrian step frequency (*r* = 0.547; *P* < 0.001; sample size: 18,538). This implies that the influence of step length on pedestrian speed is more significant than the influence of step frequency on pedestrian speed.

### Emergence of movement synchronization of pedestrians

Pedestrians spontaneously walk with the same step frequency and in phase. This familiar movement phenomenon is also known as synchronization. Previous studies (*34*, *41*, *42*, *45*) have suggested that there may exist a critical crowd density (or crowd size) at which this phenomenon occurs spontaneously. In our experiment, pedestrian foot tracking allowed to precisely capture the occurrence of synchronization. Therefore, we investigate what crowd densities are most likely to induce this phenomenon.

**Fig. 5.**
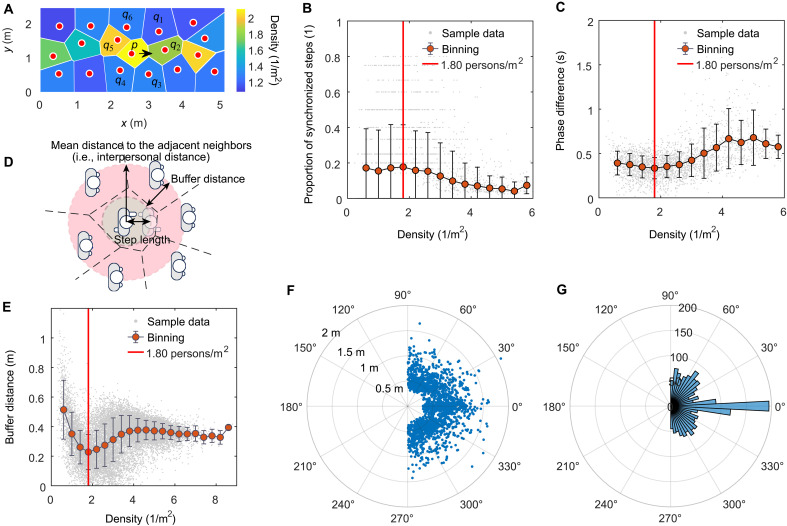
Analysis results of the movement synchronization of pedestrians. (**A**) Schematic illustration of the Voronoi diagram generated in terms of the spatial distribution of pedestrians. Here, each circle represents a pedestrian. The black arrow represents the direction of motion of the pedestrian crowd. The Voronoi cell of pedestrian *p* refers to the polygonal area surrounding pedestrian *p*. (**B**) Density-synchronization proportion relation. (**C**) Density-phase difference relation. (**D**) Schematic illustration of the buffer distance between a pedestrian and the directly adjacent neighbors after the pedestrian makes a step. (**E**) Crowd density-buffer distance relation. (**F**) Relative positions of synchronized neighbors over the collected 2076 synchronized step samples. (**G**) Distribution of the directional angles of synchronized neighbors over the 2076 collected synchronized step samples. In (B), (C), and (E), a gray point represents raw sample data. The red points represent the means after binning processing. The error bars represent the standard deviation. The red vertical line indicates that the crowd density is 1.80 persons/m^2^.

To address this problem, we analyzed the probability of the synchronization at different crowd densities. Specifically, at the starting frame of each natural step of each pedestrian, we identified the neighbors that were directly adjacent to and in front of him or her from the Voronoi diagram generated in terms of the spatial distribution of pedestrians (e.g., neighbors *q*_1_, *q*_2_, and *q*_3_ relative to pedestrian *p* in Fig. 5A). These neighbors were the nearest and directly adjacent to the pedestrian and therefore had a notable influence on the motion of the pedestrian. Next, if the starting frame *t_p_* and the duration Δ*t_p_* of this step of the pedestrian and the starting frame *t_q_* and the duration Δ*t_q_* of a step on the same foot side of an adjacent neighbor met *t_p_* − *t_q_* < *k*_1_ and Δ*t_p_* − Δ*t_q_* < *k*_2_ [where *k*_1_ and *k*_2_ represent a small relaxation quantity and are set as 0.1 s and (Δ*t_p_* + Δ*t_q_*)/16, respectively] (*34*), then the pedestrian could be approximately considered in-phase synchronized with this adjacent neighbor during this step. In the experiment, we totally detected 2076 pedestrian steps that met the above-mentioned synchronization measurement conditions. Then, we calculated the proportion of synchronized steps to total steps for each pedestrian and linked it with the mean density over the pedestrian’s steps [i.e., $\sum_{n=1}^{N_{p}}\rho \left(t_{n,s}\right)/N_{p}$ , where *N_p_* represents the number of total steps for pedestrian *p*, and *t*_*n*,s_ represents the starting moment of the *n*th step]. The obtained synchronization proportion-density relation diagram is shown in Fig. 5B. The result shows that when the crowd density is ~1.80 persons/m^2^ (i.e., the jamming density), the proportion of pedestrian synchronized steps is the highest, suggesting that synchronization is most likely to spontaneously emerge at the onset of jamming. Notably, the result also shows that in the jammed regime at high densities, as the density increases, the proportion of pedestrian synchronized steps decreases, suggesting that the probability of synchronization is small at high densities. This is because in the jammed regime at high densities, pedestrians often move one after another [i.e., stop-and-go wave phenomenon (*5*)]. That is, the movement of a pedestrian often lags behind the movement of his or her neighbors in front (i.e., a time interval exists) because only the neighbors in front move forward can the back pedestrian have enough moving space. Therefore, at high densities, the movement of pedestrians often is not synchronous as expected but one after another.

To better verify that synchronization is most likely to emerge at the jamming density of 1.80 persons/m^2^, we further calculated the phase difference (i.e., the difference in the starting moments of the nearest same-side steps between neighboring pedestrians) (*34*). The smaller the phase difference is (i.e., the smaller the difference in the start moments of the nearest same-side footsteps between neighboring pedestrians is), the more synchronized pedestrians are. We calculated the minimal phase difference with the neighbors at the start moment of each step for each pedestrian. Then, we calculated the mean of the phase difference over all steps for each pedestrian and linked this quantity with the mean density over the pedestrian’s steps. The obtained phase difference-density relation diagram is shown in Fig. 5C. The result shows that when the density is ~1.80 persons/m^2^ (i.e., the jamming density), the phase difference is the smallest, suggesting that the pedestrians are the most synchronized at the onset of jamming. The calculation of the phase difference did not involve any specified parameters and was independent of the analysis of the proportion of pedestrian synchronized steps. Therefore, this further provides plausible empirical evidence that synchronization is most likely to spontaneously emerge at the onset of jamming. Note that our study does not involve the investigation of antisynchronization. For the study of antisynchronization, the calculation and analysis of phase shift (*41*, *42*) may be required.

Notably, in previous sections, we have verified that the jamming density is exactly the flow-maximizing density. This also means that the movement synchronization of pedestrians in a general two-dimensional crowd environment is most likely to emerge at the flow-maximizing density, similar to the occurrence of the movement synchronization of pedestrians in a one-dimensional single-file crowd environment (*34*). It also should be noted that despite this similarity, the specific proportion of synchronized steps at the flow-maximizing density in this study seems to be lower than that in a one-dimensional single-file crowd environment (*34*). The main reasons may be the following: On the one hand, we adopted a stricter identification condition of synchronized steps; on the other hand, in a two-dimensional situation, pedestrians may step sideways to avoid collisions rather than synchronizing their steps, leading to a relatively low proportion of synchronized steps.

**Fig. 6.**
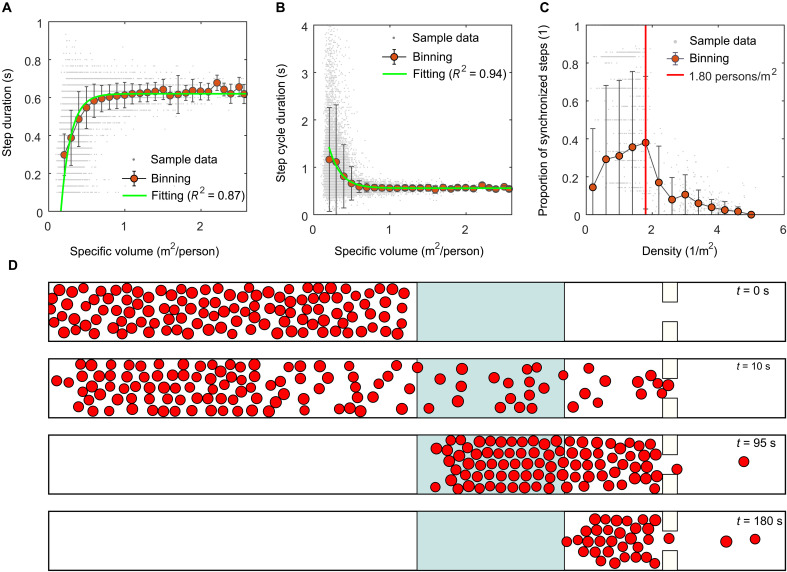
Illustration of the modeling and simulation. (**A**) Specific volume-step duration relationship. (**B**) Specific volume-step cycle duration relationship. (**C**) Density-synchronization proportion relationship obtained from the simulation. The red vertical line indicates that the density is 1.80 persons/m^2^. (**D**) Snapshots of the simulation when the crowd size and bottleneck width were 120 persons and 0.8 m, respectively. The gray area in the middle of the corridor represents the measurement area. In (A) to (C), a gray point represents raw sample data. The red points represent the means after binning processing. The error bars represent the standard deviation. In (A) and (B), the green line is the fitting result.

We additionally tested whether the finding regarding synchronization could be reflected in our numerical simulation of crowd flow (see Materials and Methods for details about the simulation model and method). As in the above analysis, we calculated the proportion of synchronized steps to total steps for each pedestrian and linked the proportion with the density in the simulation. The obtained density-synchronization proportion relationship is shown in Fig. 6C. The results show that the proportion of pedestrian synchronized steps is the highest at the jamming density (1.80 persons/m^2^), indicating that the finding that synchronization is most likely to emerge at the onset of jamming can be reflected in our numerical simulation of crowd flow.

For the detected 2076 synchronized pedestrian steps in the experiments, we also recorded the relative position of the synchronized pedestrian and neighbor and the directional angle of the synchronized neighbor (i.e., the angle between the direction of the synchronized neighbor and the desired moving direction of the pedestrian) at the starting frame of each of these 2076 synchronized step samples. Figure 5 (F and G) shows the relative positions and the distribution of the directional angles of synchronized neighbors over the collected 2076 synchronized step samples, respectively. From Fig. 5G, we can see that the number of occurrences of a 0° directional angle is obviously larger than that of 90° and 270° directional angles. This result indicates that synchronization with neighbors in front is more likely to spontaneously occur than synchronization with neighbors on the left or right; that is, front-to-back synchronization is more likely to spontaneously occur than side-by-side synchronization.

Why are pedestrians most likely to spontaneously synchronize their movements at the jamming density? To answer this question, we calculated the difference between the step length in each natural step and the interpersonal distance at the starting moment of the step (see Eqs. 3 and 6 for the calculation methods of the interpersonal distance and the step length). This difference can reflect the maintaining buffering distance between the pedestrian and the directly adjacent neighbors after the pedestrian makes a step, as illustrated in Fig. 5D. Therefore, we refer to it as buffer distance. In general, the smaller the maintaining buffer distance is after the pedestrian makes a step, the greater the risk of colliding with adjacent neighbors. We linked the buffer distance in each step of each pedestrian and the crowd density at the starting moment of the step. The resulting relation diagram is shown in Fig. 5E.

The result shows that the buffer distance-crowd density relation is nonmonotonous. The minimum buffer distance (~0.22 m) arises when the crowd density is 1.80 persons/m^2^ (i.e., at the jamming density). That is, if a pedestrian makes a step, the buffer distance between the adjacent neighbors and the body center of the pedestrian will only be 0.22 m. This means that if the neighbors stop suddenly, the body of the pedestrian will likely collide with the body of a neighbor, given that humans have a certain body thickness. However, if the pedestrian chooses to synchronize his or her stepping motion with the adjacent neighbors, the pedestrian will not only avoid potential collisions with the adjacent neighbors but also realize a stepping motion. This twofold benefit induces the pedestrian to synchronize with the adjacent neighbors. This is the reason why pedestrians are most likely to spontaneously synchronize their movements at the jamming density.

## DISCUSSION

In summary, in this study we present a large-scale crowd movement experiment that tracks the movement of pedestrian crowds through pedestrian foot tracking. Through the experiment, we first addressed what dynamical regimes pedestrian crowds can exhibit at different crowd densities. The results indicated that pedestrian crowds could exhibit three distinct dynamical regimes (free regime, slow-moving regime, and jammed regime) depending on crowd density. The transitions between them occur at crowd densities of ~0.75 and 1.80 persons/m^2^, respectively. We further investigated how pedestrians move in these different dynamical regimes. The results indicated that in the free regime, pedestrians’ movement is not constrained; in the slow-moving regime, pedestrians’ speed is constrained, but pedestrians’ movement direction in each step is not influenced; and in the jammed regime, both pedestrians’ speed and movement direction in each step are influenced. In addition, the results also indicated that in the slow-moving regime and jammed regime, pedestrians’ speed decreases exponentially with decreasing specific volume, and that in the jammed regime, the decrease in pedestrian speed results from the simultaneous decrease in pedestrian step length and step frequency, whereas in the slow-moving regime, the decrease in pedestrian speed results from a decrease in pedestrian step length and is independent of pedestrian step frequency. Last, we investigated in which dynamical regime is the movement synchronization of pedestrians most likely to spontaneously emerge. The results indicated that the movement synchronization of pedestrians is most likely to spontaneously emerge at the onset of jamming and that synchronization with neighbors in front is more likely to occur than synchronization with neighbors on the left or right.

Unlike previous studies (*2*–*9*), our experiment allowed the synchronous and precise tracking of the foot trajectories of pedestrians in two-dimensional large-scale crowds. This further enabled us to explore crowd dynamics in detail and extensively from a foot perspective. As a result, we revealed the following: (i) what dynamical regimes pedestrian crowds can exhibit at different crowd densities; (ii) how pedestrians move in these different dynamical regimes; and (iii) in which dynamical regime the movement synchronization of pedestrians is most likely to emerge.

Our study has important influences on the field of crowd dynamics. In particular, our study provides a feasible method for tracking the foot trajectories of pedestrians in two-dimensional crowds. This enables us to explore crowd dynamics in detail and extensively from a foot perspective and thus opens an avenue for the study of crowd dynamics. In addition, we report some previously unknown findings (e.g., the existence of the slow-moving regime, the characteristics of pedestrian natural steps in different dynamical regimes, and the emergence of the movement synchronization of pedestrians) into crowd dynamics; these findings greatly deepen our understanding of crowd dynamics. Notably, our study provides important inspiration for the development of pedestrian/crowd flow models in the future. Most pedestrian/crowd flow models were previously based on synchronous position update rules (*1*, *18*, *46*–*48*) (i.e., all pedestrians simultaneously update their positions and have the same time step in each step). However, as we observed in the experiment, the starting moments of the footsteps of different pedestrians might differ, and pedestrian step frequency and step duration can be dynamically adjusted in terms of the environment. It is valuable to incorporate asynchronous position updates and dynamical time steps in pedestrian/crowd flow models in future studies. Such models can eventually help to more accurately depict or predict pedestrian/crowd movements.

Similar to the locomotion of humans, the locomotion of many bipedal (quadrupedal) land animals, such as sheep, mice, and cattle (*49*–*51*), is realized by the locomotion of their two (four) feet. The presented foot tracking method can be easily extended to the foot tracking of these bipedal and quadrupedal land animals in collective motion and can be applied to the step measurement and gait analysis of these animal groups. Understanding the gait characteristics of animal groups can contribute to a better understanding of the motion of animal groups. Therefore, our study potentially has an important influence on the field of animal collective motion.

Our study has potential important applications into the field of robotics, particularly in providing quantitative support for the gait design of future bipedal humanoid walking robots. In recent years, bipedal humanoid robotics has been a highly prosperous field (*37*), and many bipedal humanoid robots (like Tesla’s Optimus robot and Boston Dynamics’ Atlas robot) have emerged. It is foreseeable that such bipedal humanoid robots are likely to integrate into human society in the near future. A core requirement for future bipedal walking robots is the ability to walk in crowd environments in a human-like manner of stepping locomotion. That is, the robots should be able to imitate the stepping locomotion of humans. Therefore, it is necessary for the designers of robots to understand the characteristics of human footsteps. In our study, the developed pedestrian foot tracking method enables to precisely measure and deeply explore the footstep characteristics of people walking in crowds. Moreover, on the basis of this method, we report many important findings regarding human footsteps (e.g., the three dynamical regimes of human walking in crowd environments and the characteristics of human step length and step frequency in different dynamical regimes). These findings can serve as important guidance for the gait design of bipedal humanoid robots (e.g., guiding the design of step length and step frequency of the robots in different dynamical regimes).

In addition to its application in the field of robotics, our study has potential practical implications for crowd flow management. Our analysis of the movement synchronization of pedestrians suggests that it can increase the movement efficiency of pedestrians. This can inform crowd managers of a potential crowd flow management strategy: making pedestrians move synchronously. This crowd flow management strategy may be feasible, because it has been reported that broadcasting rhythmic music can enhance the synchronization degree of pedestrians in a crowd (*8*). Therefore, crowd managers can broadcast rhythmic music to induce the movement synchronization of pedestrians and use such deliberate synchronization stimuli to improve the movement efficiency of pedestrians and accelerate the flow of pedestrian crowds. This crowd flow management strategy can potentially be applied to crowd flow management in some public spaces (i.e., subway stations) to accelerate the flow of pedestrian crowds in these spaces.

The movement synchronization of pedestrians is a double-edged sword. As mentioned above, movement synchronization can enhance the movement efficiency of pedestrians. However, it was also regarded as a possible trigger of some crowd disasters, such as the wobbling accident of the London Millennium Bridge (*35*, *36*). Although there are still some controversies about whether synchronization is the cause or consequence of the accident (*52*), in general, the more synchronized pedestrians themselves are, the greater the dynamic stimulus to the building is (because of simultaneous foot forces from pedestrians) and the greater the possibility of the vibration of the building is. Our study reveals that the movement synchronization of pedestrians is most likely to spontaneously emerge at the critical density between the slow-moving regime and the jammed regime. This finding implies that in terms of crowd flow management, controlling the density of crowd flow to deviate from this critical density may be a potential countermeasure for reducing the probability of such crowd synchronization–induced building vibration. Notably, crowd synchronization–induced building vibration is related not only to the movement of pedestrian crowds but also to the building structure itself. Therefore, the mechanism of crowd synchronization–induced building vibration is very intricate and remains to be further explored. In future work, it will be necessary to combine crowd movement and building structure vibration experiments to better explore the emergence of crowd synchronization–induced building vibration.

The main limitations of this study are as follows: First, we only explored the dynamics of pedestrian crowds in the most common unidirectional movement. In future work, it will be necessary to explore the dynamics of pedestrian crowds through the foot perspective in more complex multidirectional movement scenarios. In addition, because the main focus of this study is to explore pedestrian crowd behavioral characteristics and phenomena that are difficult to clearly capture via pedestrian head tracking but can be clearly captured by pedestrian foot tracking, we have not investigated the lane formation, stripe formation, and arching and clogging phenomena in this study (*9*, *10*, *19*, *53*). In future work, further exploration of these self-organized phenomena from the foot perspective is necessary. Second, the proposed foot tracking method can be applied for high-density and various typical movement scenarios in laboratory experimental contexts. However, for this method, there are still some application limitations in real-life contexts because in real life, the resources of elevated pedestrian corridors constructed with glass floors are scarce. However, with the increase in the number of modern glass skywalks throughout the world, it is very promising to apply this method in real-life contexts. Third, the experiment involves only young participants. Some results may differ from the results when participants of different ages are involved. For example, if older pedestrians are involved, the resulting critical density between the free regime and the slow-moving regime may be lower than 0.75 persons/m^2^, because old people usually tend to maintain a larger distance for safety than young people do (*39*). Fourth, it is worth further exploring the link between the three revealed dynamical regimes and the pedestrian Level of Service in future work. In the pedestrian Level of Service framework (*54*, *55*), Level A corresponds to a full and rigorous unconstrained pedestrian movement state, which seems to be equivalent to the free regime defined in this study. Level F corresponds to a severely constrained pedestrian movement state that decreases the flow rate, which seems to be equivalent to the jammed regime defined in this study. Therefore, the slow-moving regime defined in this study may correspond to Levels B to E. Fifth, in this study, the identification of the boundary between the free regime and the slow-moving regime is based on the observation of speed change trend, as in many previous studies (*5*, *43*). It is necessary to deduce a quantitative analytic method or define a unified criterion to identify this boundary in future work. A potential scheme is to introduce a small speed change threshold, like the identification of the boundary between the stop state and the moving state of pedestrians in the empirical analysis of pedestrian movements (*27*, *34*, *43*). That is, when the speed deviation from the free speed exceeds a small speed change threshold (e.g., 0.01 m/s), pedestrians can be regarded in an unfree regime. In future work, further studies will be needed to define or determine the optimal or acceptable value of this speed change threshold. Sixth, it is possible that pedestrians synchronize by chance. This may lead to some statistical errors in the analysis of the synchronization (their influence on the final results may be very slight because of the large total sample size of synchronized steps). In future work, it is necessary to further improve the experimental and analysis methods to solve this issue. Last, this study did not investigate the antisynchronization phenomenon of pedestrians. The emergence of antisynchronization can be identified through the analysis of the phase shift of pedestrian footsteps (*41*, *42*). In future work, it is necessary to systematically investigate the emergence of antisynchronization and synchronization.

## MATERIALS AND METHODS

### Experimental design

The experiment was carried out in September 2022 at Sichuan University in China. Unlike many previous large-scale crowd movement experiments, in which the motion tracking of pedestrian crowds was based on pedestrian head tracking (*2*–*9*), in our experiment, we tracked the movement of pedestrian crowds through pedestrian foot tracking. Figure 1 displays the principle of the developed tracking method. Specifically, pedestrian foot tracking was realized through an elevated pedestrian corridor. The height of the corridor to the ground and the total length and width of the corridor were 1.27, 25.02, and 2.40 m, respectively. At the two ends of the corridor, there were stairs to the ground. The whole platform was assembled of many desk-like units. Therefore, the size, height, shape, and layout of the platform could be flexibly adjusted. The floor of the middle segment of the corridor was a whole piece of specially designed toughened laminated glass plate with a length of 5.50 m, a width of 2.40 m, and a thickness of 0.06 m (note that such a glass plate was sufficiently tough to support pedestrian walking and was often used to build sightseeing glass skywalks on canyons and skyscrapers). That is, the floor of the middle segment of the corridor was fully transparent. This key design allowed us to clearly and simultaneously monitor the pedestrians’ foot motion from the ground with a bottom-up viewing angle when pedestrians walked through this area, as shown in Fig. 1C.

We conducted crowd movement experiments in the corridor using pedestrian crowds of different densities. A total of 120 participants (60 males and 60 females; mean age, 20.37 ± 1.95 years; mass, 61.33 ± 9.18 kg; body radius, 0.21 ± 0.02 m) recruited from university students participated in the experiments. The experiments complied with all relevant ethical regulations, including obtaining informed consent from all participants. Before the formal experiment, we conducted a couple of pretest runs with all the participants in the corridor. To regulate the crowd density in the corridor, we set a bottleneck at 3.6 m from the transparent observation area on one side of the transparent observation area. Then, we performed 40 runs of experiments using different crowd sizes and bottleneck widths, including using 20, 30, 40, 50, 60, 70, 80, 90, 100, 110, and 120 participants in the case of a bottleneck width of 2.4 m, as well as using bottleneck widths of 2.2, 2.0, 1.8, 1.6, 1.4, 1.2, 1.0, 0.8, 0.7, 0.6, 0.5, and 0.4 m in the case of 120 participants. Table 1 lists the detailed runs in each case. In each run, the participants were initially distributed uniformly on one side of the corridor. After giving the whistle to start the experiment, the participants started to walk in a natural way along the corridor, passed the transparent observation area and bottleneck, and reached the other side of the corridor. Each run ended after all the participants had passed the bottleneck. In the experiments, the participants were asked to wear shoe covers on two feet. At the bottom of the shoe covers on two feet, we attached red and green tracking markers, respectively, as shown in Fig. 1C. This enabled us to observe the motion of pedestrians’ two feet more clearly in the experiments. We set a high-resolution camcorder under the transparent glass floor of the observation area to record the pedestrians’ foot motion with a bottom-up viewing angle during the experiments. The resolution and frame rate of the camcorder were set to 1920 by 1080 pixels and 30 frames per second, respectively.

**Table 1.** Experiment runs under different crowd sizes and bottleneck widths.

Number of pedestrians	Bottleneck width (m)	Number of runs	Mean density (persons/m^2^)
10	2.4	1	0.53
20	2.4	7	0.71
30	2.4	4	0.95
40	2.4	4	1.11
50	2.4	3	1.34
60	2.4	2	1.40
70	2.4	1	1.61
80	2.4	1	1.71
90	2.4	1	1.96
100	2.4	1	2.07
110	2.4	1	2.11
120	2.4	1	2.28
120	2.2	1	2.24
120	2.0	1	2.38
120	1.8	1	2.73
120	1.6	1	2.78
120	1.4	1	2.81
120	1.2	1	3.14
120	1.0	1	3.37
120	0.8	1	3.64
120	0.7	1	3.78
120	0.6	1	4.25
120	0.5	1	4.30
120	0.4	2	4.36

### Trajectory extraction

Given that the video image distortion was relatively large at the left and right far-ends of the transparent observation area, we used only the area of 4.20 m by 2.40 m at the middle of the transparent observation area as the actual data collection area. We extracted the two-dimensional trajectory data of the foot motion of the pedestrians in the data collection area from the obtained video clips using a mean-shift clustering algorithm and transformed them from the video space to the real physical space using a linear transformation method (*56*). The maximal measurement error of the trajectories was ~0.11 m, which mainly occurred at the left and right far-ends of the data collection area and resulted from the change in the height of pedestrian feet during the walking.

Note that in the experiment, we also set tracking markers on the pedestrians’ heads and a camcorder overhead in the measurement area to record the pedestrians’ head motions, as shown in Fig. 1D. However, the extracted pedestrian head motion data were not directly used in this study. This is because we obtained the coordinates of each pedestrian’s two feet at each moment. Therefore, we could take the center of the two feet (i.e., the mean position of the two feet) of a pedestrian as the pedestrian’s center of mass and correspondingly use the motion trajectory of this point to represent the motion trajectory of the pedestrian.

### Measurement methods for motion parameters

#### Starting and ending moments of each natural step

To identify the starting and ending moments of each natural step of pedestrians, we calculated the instantaneous speed of each foot of each pedestrian at each frame using Eq. 1

$$v\left(t\right)=\frac{|X\left(t+\Delta t/2\right)-X\left(t-\Delta t/2\right)|}{\Delta t/30}$$ (1)

where *v*(*t*) and **X**(*t*) are the instantaneous speed and coordinate of a single foot at the frame moment *t*, respectively. Δ*t* is the time interval (six frames are used here), and the number 30 is the frame rate of the video recordings.

Figure 1E shows an example of the variation in the instantaneous speed of each foot of a pedestrian with time when the pedestrian passed through the transparent observation area. The starting and ending moments of each natural step of the pedestrian can be identified through this time-speed diagram. Specifically, when a footstep starts, the foot lifts from the ground and thus changes from a stop regime to a motion regime. When the instantaneous speed of the foot was less than 0.1 m/s, the foot could be regarded as in a stop regime (*27*, *34*, *43*) [note that reducing this speed threshold value could lead to the misinterpretation of some data noise (i.e., those tiny fluctuations without regularity below the threshold line in Fig. 1E) to be pedestrian footsteps, thus resulting in fake pedestrian footstep samples]. Therefore, if *v* (*t*_*n*,s_ − 1) < 0.1 m/s and *v* (*t*_*n*,s_) ≥ 0.1 m/s, then the frame moment *t*_*n*,s_ can be identified as the starting moment of the pedestrian’s *n*th step. When this footstep ends, the foot will return to the ground and thus change from a motion regime to a stop regime. Therefore, if *v* (*t*_*n*,e_ − 1) ≥ 0.1 m/s and *v* (*t*_*n*,e_+1) < 0.1 m/s, then the frame moment *t*_*n*,e_ can be identified as the ending moment of this footstep. Likewise, the starting moment of the pedestrian’s *n* + 1th footstep can be identified if *v* (*t*_*n*+1,s_ − 1) < 0.1 m/s and *v* (*t*_*n*+1,s_) ≥ 0.1 m/s.

#### Density and interpersonal distance

The local density ρ*_p_* (*t*) of pedestrian *p* at the frame moment *t* can be calculated using Eq. 2

$$\rho_{p}\left(t\right)=\frac{1}{A_{p}\left(t\right)}$$ (2)

where *A_p_*(*t*) denotes the area of the Voronoi cell of pedestrian *p* in the Voronoi diagram generated in terms of the spatial distribution of the pedestrians in the *t*th frame in the measurement area, as illustrated in Fig. 5A. Namely, we used the Voronoi diagram method (*57*, *58*) to calculate the density.

The interpersonal distance *D_p_*(*t*) of pedestrian *p* at the frame moment *t* can be calculated using Eq. 3

$$D_{p}\left(t\right)=\frac{\sum_{q=1}^{Q_{p}}|X_{p}\left(t\right)-X_{q}\left(t\right)|}{Q_{p}\left(t\right)}$$ (3)

where **X***_p_*(*t*) and **X***_q_*(*t*) denote the coordinates of pedestrian *p* and directly adjacent neighbor *q* at the frame moment *t*, respectively. *Q_p_*(*t*) is the number of neighbors directly adjacent to pedestrian *p* at the frame moment *t*. Note that the directly adjacent neighbors refer to these neighbors that were directly adjacent to pedestrian *p* from the Voronoi diagram generated in terms of the spatial distribution of pedestrians (e.g., neighbors *q*_1_ to *q*_6_ relative to pedestrian *p* in Fig. 5A).

#### Flow speed and flow rate of pedestrians

The flow speed *v_p_*(*t*) of pedestrian *p* at the frame moment *t* and the corresponding flow rate *f_p_*(*t*) can be calculated using Eqs. 4 and 5, respectively

$$v_{p}\left(t\right)=\frac{|x_{p}\left(t\right)-x_{p}\left(t+\Delta t\right)|}{\Delta t/30}$$ (4)

$$f_{p}\left(t\right)=\rho_{p}\left(t\right)\times v_{p}\left(t\right)$$ (5)

where *x_p_*(*t*) denotes the longitudinal coordinate of pedestrian *p* at the frame moment *t*, i.e., the longitudinal component of pedestrian coordinate **X***_p_* (*t*). Δ*t* is the time interval (here, Δ*t* is set as the real interval of each natural step, i.e., *t*_*n*,e_-*t*_*n*,s_). The number 30 is the frame rate of the video recordings. Note that the speed *v_p_* (*t*) here is the pedestrian flow speed (*4*), i.e., the moving speed along the desired flow direction of pedestrians, which can more essentially reflect the actual effective flow of pedestrian toward the target.

#### Step length, step frequency, stop duration, and directional angle of pedestrians in each natural step

The step length *L_p_*(*n*), step frequency *F_p_*(*n*), stop duration *S_p_*(*n*), and directional angle θ*_p_*(*n*) of pedestrian *p* in the *n*th step can be calculated using Eqs. 6 to 9, respectively.

$$L_{p}\left(n\right)=\left|X_{p}\left(t_{n+1,s}\right)-X_{p}\left(t_{n,s}\right)\right|$$ (6)

$$F_{p}\left(n\right)=\frac{30}{t_{n+1,s}-t_{n,s}}$$ (7)

$$S_{p}\left(n\right)=\frac{t_{n+1,s}-t_{n,e}}{30}$$ (8)

$$\begin{array}{c} \theta_{p}\left(n\right)={\text{cos}}^{-1}\left\{\;\begin{array}{c} \frac{\left[X_{p}\left(t_{n,e}\right)-X_{p}\left(t_{n,s}\right)\right]\cdot E}{|X_{p}\left(t_{n,e}\right)-X_{p}\left(t_{n,s}\right)\|E|} \end{array}\;\right\} \end{array}$$ (9)

where *t*_*n*,s_ and *t*_*n*,e_ represent the starting and ending moments of the *n*th step of pedestrian *p*, respectively. The number 30 is the frame rate of the video recordings. **E** represents the desired moving direction of the pedestrian, i.e., the direction parallel to the corridor.

### Methods of additional modeling and simulation

#### Basic model

The adopted basic simulation model was the social force model (*18*). In the social force model, the pedestrian’s movement is governed by the acceleration equation

$$\begin{array}{c} m_{p}\frac{dv_{p}}{\mathrm{dt}}=m_{p}\frac{v_{p}^{0}\left(t\right)e_{p}^{0}\left(t\right)-v_{p}\left(t\right)}{\tau_{p}}+\sum_{q\left(\neq p\right)}f_{\mathrm{pq}}+\sum_{W}f_{\mathrm{pw}} \end{array}$$ (10)

The first term at the right-hand side of the equation represents the attractive force from the target. In this term, *m_p_* represents the mass of pedestrian *p*, $v_{p}^{0}$ represents the pedestrian’s desired speed, $e_{p}^{0}$ represents the pedestrian’s desired direction, and τ is the relaxation time.

The second term at the right-hand side of the equation represents the interaction force between the pedestrian and neighbors, which is calculated by

$$\begin{array}{c} f_{\mathrm{pq}}=A_{p}\text{exp}\left(\frac{r_{\mathrm{pq}}-d_{\mathrm{pq}}}{B_{p}}\right)n_{\mathrm{pq}}+\mathrm{kg}\left(r_{\mathrm{pq}}-d_{\mathrm{pq}}\right)n_{\mathrm{pq}}+\kappa g\left(r_{\mathrm{pq}}-d_{\mathrm{pq}}\right)\;\Delta v_{\mathrm{qp}}^{t}t_{\mathrm{pq}} \end{array}$$ (11)

where *A_p_* exp.((*r_pq_* − *d_pq_*)/*B_p_*)**n***_pq_* represents the psychological repulsive force between pedestrians *p* and *q*. *A_p_* and *B_p_* are constants, and *r_pq_* is the sum of the body radius of pedestrians *p* and *q*. *d_pq_* is the distance of centers of mass between pedestrians *p* and *q*. **n***_pq_* = (*n_pq_*^1^, *n_pq_*^2^) = (**r***_p_* − **r***_q_*)/*d_pq_* represents the normalized vector pointing from pedestrian *q* to *p*. *kg*(*r_pq_* − *d_pq_*)**n***_pq_* and κ*g*(*r_pq_* – *d_pq_*) $\Delta v_{\mathrm{qp}}^{t}$**t***_pq_* represents the body force and sliding friction force, respectively. They appear when pedestrians *p* and *q* touch each other (i.e., *d_pq_* < *r_pq_*). *g*(*x*) equals to *x* when the touch arises; otherwise, it equals to 0. **t***_pq_* = (−*n_pq_*^2^, *n_pq_*^1^) represents the tangential direction. $\Delta v_{\mathrm{qp}}^{t}$ = (**v***_q_* – **v***_p_*)·**t***_pq_* represents the velocity difference between pedestrians *p* and *q*. *k* and κ are constants.

The third term at the right-hand side of the equation represents the interaction force between the pedestrian and the boundary. Similar to the calculation of the interaction force between the pedestrian and the neighbor, the interaction force between the pedestrian and the boundary can be calculated by

$$\begin{array}{c} f_{\mathrm{pw}}=A_{p}\text{exp}\left(\frac{r_{p}-d_{\mathrm{pw}}}{B_{p}}\right)\;n_{\mathrm{pw}}+\mathrm{kg}\left(r_{p}-d_{\mathrm{pw}}\right)n_{\mathrm{pw}}+\kappa g\left(r_{p}-d_{\mathrm{pw}}\right)\left(v_{p}\cdot t_{\mathrm{pw}}\right)t_{\mathrm{pw}} \end{array}$$ (12)

where *d_pw_* represents the distance between pedestrian *p* and boundary *w*, **n***_pw_* represents the direction perpendicular to the boundary, and **t***_pw_* represents the tangential direction.

#### Model calibration

We used the data obtained from our experiment to calibrate the model. The pedestrian masses and body radii could be collected from the experimental participants (see fig. S2 for the distributions of the collected pedestrian masses and body radii). Accordingly, the pedestrian mass and body radius in the model could be generated in terms of the distributions of the pedestrian masses and body radii. Pedestrians would change their desired speed *v*^0^ in terms of their specific volume η, as suggested by the specific volume-speed relation obtained from our experiment (see Fig. 2D). Therefore, the desired speed *v*^0^ of pedestrians could be determined by the specific volume-speed relationship $v^{0}=1.16\left[1-e^{-4.45\left(\eta -0.23\right)}\right]$ . The main model parameters that had to be calibrated were *A_p_*, *B_p_*, *k*, κ, and τ. Many methods exist for the calibration of these parameters (*59*–*61*). Here, we applied a differential evolution method (*61*) to estimate the values of these parameters using the pedestrian trajectory data obtained from our experiment. The estimated values of *A_p_*, *B_p_*, *k*, κ, and τ were 42.17 N, 0.073 m, 95,129.84 kg/s^2^, 285066.61 kg/m·s, and 0.31 s, respectively.

#### Position update scheme of pedestrians

When walking in crowds, pedestrians adaptively adjust their step frequency, step cycle duration, and step duration in terms of the real-time specific volume (note that the step cycle duration is the reciprocal of the step frequency and is not equivalent to the step duration, as illustrated in Fig. 1E). By linking the step cycle duration and the step duration for each of the detected pedestrian steps in the experiment with the specific volume at the starting moment of the step, we found that the specific volume (η)-step duration (*Ts*) relationship could be approximately described as $\mathrm{Ts}=0.62\left[1-e^{-7.79\left(\eta -0.16\right)}\right]$ (*R*^2^ = 0.87; sample size: 25), whereas the specific volume (η)-step cycle duration (*Tsc*) relationship could be approximately described as $\mathrm{Tsc}=3.19e^{-6.49\eta }+0.56$ (*R*^2^ = 0.94; sample size: 25), as shown in Fig. 6 (A and B). Using the obtained specific volume-step cycle duration and specific volume-step duration formulas, we could estimate the step frequency, step cycle duration, and step duration at different specific volumes. Once estimated, we could update the position for the *n*th step of pedestrian *p* by Eq. 13 and calculate the starting moment of the next step by Eq. 14

$$r_{p}\left(t_{n,s}+\mathrm{Dt}\right)=r_{p}\left(t_{n,s}\right)+\mathrm{Dt}\cdot v_{p}\left(t_{n,s}\right)$$ (13)

$$t_{n+1,s}=t_{n,s}+\mathrm{Tsc}$$ (14)

where **v***_p_*(*t*_*n*,s_) represents the velocity vector estimated by the social force model. *Dt* represents the duration of pure foot motion during a step cycle, and its value is given by the following

$$\mathrm{Dt}=\left\{\;\begin{array}{cc} \mathrm{Ts} & \text{if}\;\eta \;>0.16\;\text{and}\;\mathrm{Ts}\;\leq \;\mathrm{Tsc}\;\\ \mathrm{Tsc} & \text{if}\;\eta \;>0.16\;\text{and}\;\mathrm{Ts}\;>\;\mathrm{Tsc}\\ 0.01 & \text{if}\;\eta \;\leq \;0.16 \end{array}\right.$$ (15)

where the number 0.16 represents the critical specific volume when the step duration decreases to zero. Note that it is possible that a pedestrian’s specific volume in the simulation was smaller than this theoretical value. In this case, *Dt* was set to be a small relaxation value close to zero (i.e., 0.01) to prevent the occurrence of a deadlock.

Notably, because the step cycle duration, step duration, and step frequency (i.e., the reciprocal of the step cycle duration) of pedestrians were adaptively adjusted in terms of their real-time specific volume, the position updates of pedestrians in each step were not simultaneous, but rather asynchronous, as in real life. Therefore, the presented model was more human-like than many previous models based on synchronized parallel or sequential pedestrian position update (*1*, *18*, *46*–*48*).

#### Simulation

On the basis of the calibrated model and the proposed pedestrian position update scheme, we conducted a crowd movement simulation in a virtual corridor with the same layout and size as the real corridor in the experiment. We performed a total of 40 runs of simulations using different crowd sizes and bottleneck widths as the setup in our experiment. In each run, pedestrians were initially set to be randomly distributed at the waiting area at one side of the measurement area with a random beginning step phase. The pedestrian masses and body radii were generated in terms of the distributions of the collected values of these parameters. Note that the generated pedestrian body diameter could be greater than the minimal bottleneck width of 0.4 m. In the experiment, pedestrians with a body diameter greater than 0.4 m could pass the bottleneck width of 0.4 m, because the human body is flexible and real pedestrians can reduce their body size by turning their bodies sideways. However, in the social force model, pedestrians are circular; thus, their body size cannot be reduced by turning their body. Therefore, in the runs with the bottleneck width of 0.4 m, the pedestrian body diameter automatically decreased to 0.4 m when the pedestrian reached the bottleneck to ensure that the pedestrian could pass the bottleneck. In addition, the desired direction $e_{i}^{0}$ of pedestrians was set parallel to the corridor in the runs without a bottleneck. In the runs with a bottleneck, $e_{i}^{0}$ was set parallel to the corridor when pedestrians were in the measurement area given that the measurement area was far from the bottleneck, whereas $e_{i}^{0}$ was set to point to the bottleneck after pedestrians walked out of the measurement area. Each run ended when all pedestrians walked out of the bottleneck.

Figure 6D shows snapshots of the simulation when the crowd size and bottleneck width were 120 persons and 0.8 m, respectively. Figure 3A shows the specific volume-speed, density-flow rate, and density-speed relationship diagrams obtained from the simulation. Figure 6C shows the density-synchronization proportion relationship diagram obtained from the simulation (note that the calculation methods of the density, speed, flow rate, and synchronization proportion in the simulation were the same as those in the preceding analysis of the experimental results). Figure 3A shows that the profiles of the specific volume-speed, density-flow rate, and density-speed relationship diagrams obtained from the simulation were similar to those obtained from the experiment. The free regime, slow-moving regime, and jammed regime could be observed. Moreover, Fig. 6C shows that the obtained density-synchronization proportion relationship diagram could reflect that the movement synchronization of pedestrians was most likely to emerge at the flow-maximizing density, similar to the findings in the experiment. These results indicated that the quality and performance of the presented model were acceptable.

### Source of the datasets used to additionally verify the three dynamical regimes

The datasets used to additionally verify the three dynamical regimes included datasets of a unidirectional crowd flow, a bidirectional counter crowd flow, a bidirectional cross-crowd flow, and a four-directional cross-crowd flow (*4*, *62*–*63*). These datasets are public and can be found in the Pedestrian Dynamics Data Archive: http://ped.fz-juelich.de/db/.
