# Supplementary material for: Unraveling human crowd dynamics through the foot tracking of pedestrians
Source: Sci Adv. 2025 Jun 25;11(26):eadw2688. doi: 10.1126/sciadv.adw2688 (PMC12189950; doi:10.1126/sciadv.adw2688)
Supplement: Supplementary file 1 — Figs. S1 and S2 [file sciadv.adw2688_sm.pdf]

Science Advances
AAAS

Supplementary Materials for

Unraveling human crowd dynamics through the foot tracking of pedestrians

Yi Ma *et al.*

Corresponding author: Yi Ma, yima23-c@my.cityu.edu.hk

Sci. Adv. **11**, eadw2688 (2025)
DOI: 10.1126/sciadv.adw2688

This PDF file includes:

Figs. S1 and S2

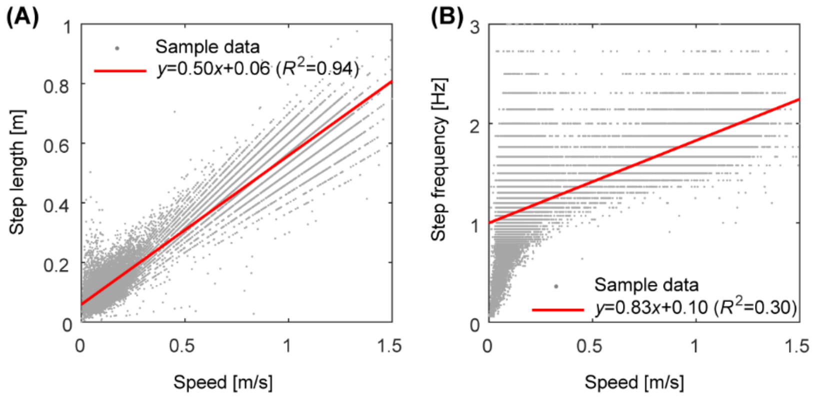

Fig. S1.

Correlations between pedestrian movement parameters. (A) Speed versus step length. **(B)** Speed versus step frequency.

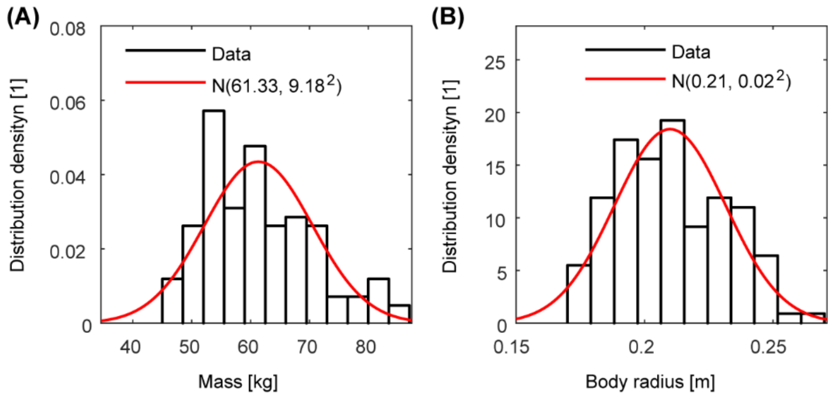

Fig. S2.

Distributions of the collected experimental participants' masses and body radii. (A) Mass distribution. **(B)** Body radius distribution.
